# Revisiting Fitch and Hauser's Observation That Tamarin Monkeys Can Learn Combinations Based on Finite-State Grammar

**DOI:** 10.3389/fpsyg.2021.772291

**Published:** 2021-11-29

**Authors:** Shigeru Miyagawa

**Affiliations:** Department of Linguistics and Philosophy, Massachusetts Institute of Technology, Cambridge, MA, United States

**Keywords:** Chomsky hierarchy, tamarin, finite-state grammar, phrase-structure grammar, alarm calls, frontal operculum, Broca's area, putty-nosed monkey

## Introduction

In a groundbreaking work, Fitch and Hauser (2004) compared artificial grammar learning between human and cotton-top tamarins (*Saguinus oedipus*) using finite-state grammar (FSG) and phrase-structure grammar (PSG) types. They found that while humans are able to learn both grammar types, the tamarin monkeys could only learn combinations based on FSG. FSGs process linearly ordered strings whose structure resorts to strictly adjacent steps. Examples of FSGs are A^n^ and (AB)^n^, where *n* indicates the number of times A and AB are repeated, and A^n^B^m^, where *n* ≠ *m*. On the other hand, PSGs are not limited to adjacency. This allows PSGs to match the number of units repeated in each series generated, as in the sequence A^n^B^n^, where the number of A's matches the number of B's (Balari et al., 2011; Longa, 2013). The non-adjacent relations in PSGs are made possible by hierarchical structures that relate items at a distance. Since PSGs require hierarchical structure, F&H conclude that while humans can generate them, tamarins cannot, thus limiting their system to sequences based strictly on adjacent dependency, that is, FSG. There is no doubt that human language requires a grammar more powerful than FSG (Chomsky, 1956, 1959). In this article, I will take up F&H's assumption that their experiment showed that the tamarin monkeys are capable of learning sequences based on FSG. While their stimuli appear to approximate an FSG, in reality they do not, except trivially. Hence, their conclusion that tamarins are capable of FSG is at best weak. This casts doubt on using the Chomsky hierarchy for describing the learning behavior of nonhuman primates. Furthermore, unlike humans, who are exposed continuously to natural speech that requires a grammar more powerful than FSG, monkeys in nature are never exposed to verbal behavior that reflects FSG in any meaningful sense. It would therefore be surprising if they exhibit mastery of FSG combinations, which are entirely outside their natural experience.

## Fitch and Hauser's Experiments

In F&H's experiments, the stimuli were composed of two categories: in one category are female utterances artificially synthesized into discrete consonant-vowel syllables (***pa***, ***li***, ***mo***, ***nu***, ***ka***…), and in the other are male utterances similarly synthesized into discrete syllables that differed from the female syllables (*ba, di, yo, tu, no …*). The male/female syllables also differ distinctly in pitch as well as in other acoustic variables. For FSG, a syllable from one category (A) is followed by a syllable from the other category (B) (e.g., ***no** li*). Similar A-B combinations with different syllables were played in sequence, A-B, A-B, A-B. This is a straightforward Markovian system in which a given automaton is carried from one finite state [n] to the next state [n+1]. F&H demonstrated that cotton-top tamarin monkeys can learn (AB)^n^. For PSG, three syllables from one category were played, followed by three syllables from the other category: A-A-A-B-B-B. FSG cannot generate this structure without incurring significant cost because the operation depends on non-adjacent information. As F&H note, the first “A” predicts the occurrence of the final “B”, and the second “A” predicts the second “B”, and the final “A” predicts the first “B.” This combination reflects a formal grammar higher on the Chomsky hierarchy (Chomsky, 1956)—PSG, which requires hierarchical relations. The tamarin's ability to learn fails completely when presented with the sequence A^n^B^n^ generated by PSG. On the other hand, humans readily learn both types of sequences. F&H conclude that the crucial difference is that while humans can generate hierarchical structures that can create non-adjacent dependencies, tamarins are unable to do so, thus limiting their system to adjacent relations. This is an important study for distinguishing human and nonhuman primate learning abilities. The question is, what precisely is the difference? While humans are capable of learning combinations based on a formal grammar more powerful than FSG, I will take issue with F&H's assumption that what we see with tamarins is an ability to learn combinations based on FSG.^1^

Their so-called FSG is the binary combination, AB. This is FSG only trivially. As I will show, in nature, monkeys are exposed most commonly to a combination of one, but we do see instances of a dual combination. The point is that we don't see anything that exceeds two, which would be surprising if the monkeys are capable of FSG.^2^

## Questioning Whether Tamarins Can Learn Combinations Based on FSG

In natural settings, nonhuman primate calls are typically isolated units. The alarm calls of the vervet monkey (Struhsaker, 1967; Seyfarth et al., 1980a,b) is one such system. Vervet monkeys (*Chlorocebus pygerythrus*) give a distinct call when they encounter a leopard, another when they see an eagle, and a third when they come across a snake. They never combine two calls to produce a new call. This is what Miyagawa and Clarke (2019) call the *System of One*, and it is the predominant system for alarm calls. This leads to the question, what do we make of the demonstrated ability of tamarins to be able to learn A-B sequences?

## System of Two

Some Old World monkeys such as the Guenons of Africa produce utterances that Miyagawa and Clarke (2019), based on much prior research, analyzed as being composed of two items. A key observation is that this binary system is just that—binary. One never sees a combination that begins A, B, then goes to C, or returns to A. This is a fundamentally different behavior from what F&H would predict, because a sequence of A-B-C or A-B-A is possible in FSG. Below, I will demonstrate this binary nature using the system employed by putty-nosed monkeys (*Cercopithecus nictitans*).

There are two main alarm calls associated with the putty-nosed monkeys, *pyow* (=P), which is a general alarm call, and *hack* (=H), which is typically used in the presence of eagles. The putty-nosed monkeys also produce *pyow-hack* combinations consisting of a number of *pyow*s followed by a number of *hacks*. While the individual *pyow*s and *hacks* are alarm calls, the *pyow-hack* sequences relate to group movement. Using playback experiments, Arnold and Zuberbühler (2006a,b, 2008, 2012, 2013) demonstrated that the overall length of the sequence is statistically related to the distance traveled by the group; the number of *pyow*s and *hack*s within the equal-length sequences did not affect the distance. Thus, the researchers observed similar behavior when they played back PPPHHH, PHHHHH, and other P-H combinations of the same length.

Schlenker et al. [(2016): 33] point out that the different *pyow-hack* sequences of the same length are phonologically complex, but lexically simple. They are phonologically complex due to the various numbers of *pyow*s and *hack*s (see also Mitani and Marler, 1989). The sequences are lexically simple because they are associated with comparable distance traveled, regardless of the number of actual *pyow*s and *hack*s. How can we capture both the phonological complexity and the lexical simplicity of these sequences? Looking at the different possibilities, there are two compartments, one for *pyow*s, the other for *hack*s, as shown in Table 1.

**Table 1:** Dual-compartment frame (Miyagawa and Clarke, 2019).

***pyow* ^**+**^**	***hack* ^**+**^**
1	2

Each compartment may contain a varying number of *pyows* or a varying number of *hacks*.^3^ Crucially, we never find a sequence such as PHP (Arnold and Zuberbühler, 2012), because this sequence would require more than two compartments. On the FSG view of monkey learning behavior, we would predict that PHP is possible, contrary to fact.^4^

Other systems that Miyagawa and Clarke (2019) explore have the same dual-compartment character. The dual-compartment frame can trivially be modeled by FSG, but it is by no means FSG in the standard sense in that there is no operation of any kind that can potentially lead to strings of infinite length.

## Discussion

While F&H used the (AB)^n^ stimulus under the assumption that this models FSG, I suggest that what F&H demonstrated for tamarins was that they are capable of learning binary combinations, which occur in natural settings. In fact, F&H [(2004): 379] entertain the possibility that “tamarins fail the PSG because their ability to differentiate successive items is limited to runs of two.” They reject this idea because they tested A-A-B-B along with A-A-A-B-B-B, and tamarins failed to learn both sequences. However, A-A-B-B sequence cannot easily fit into the dual-compartment frame because for each A, there is B. This kind of relation is expressed by a hierarchical structure, as F&H themselves note. This, in turn, casts doubt on applying formal grammar based on the Chomsky hierarchy for distinguishing learning behavior of nonhuman primates from that of humans. The learning behavior of nonhuman primates does not appear susceptible even to the simplest formal grammar (FSG) on the hierarchy.

There is also neuroanatomical evidence for the idea that the (AB)^n^ sequence as used by F&H does not implicate FSG. Friederici et al. (2006) (see also Friederici et al., 2012) demonstrated that the PSG sequence, A^n^B^n^, similar to the stimulus created by F&H, activates Brodmann area 44 of the Broca's area and the frontal operculum.^5^ In contrast, the “FSG” sequence of (AB)^n^ only recruits the frontal operculum. The frontal operculum is a phylogenetically older part of the brain than the Broca's area (Sanides, 1962). As Zaccarella and Friederici (2015a,b,c) note, one of its functions is apparently to create (AB) combinations, which we see in monkeys (Sanides, 1962) and in humans.^6^ The Broca's area and the frontal operculum each has a unique functional, anatomical and molecular brain architecture (Sanides, 1962; Amunts et al., 1999, 2010; Zilles and Amunts, 2009). It is Broca's region of the brain that is recruited for the complex PSG-based sequence, which has a hierarchical structure.^7^ From this perspective, the AB sequence that F&H showed to be learnable by tamarins need not be understood as an indication of their ability to learn combinations based on FSG. Rather, it fits the binary combination that models the dual-compartment frame arguably activated in the frontal operculum.

## Author Contributions

The author confirms being the sole contributor of this work and has approved it for publication.

## Conflict of Interest

The author declares that the research was conducted in the absence of any commercial or financial relationships that could be construed as a potential conflict of interest.

## Publisher's Note

All claims expressed in this article are solely those of the authors and do not necessarily represent those of their affiliated organizations, or those of the publisher, the editors and the reviewers. Any product that may be evaluated in this article, or claim that may be made by its manufacturer, is not guaranteed or endorsed by the publisher.

## Footnotes

^1^Studies have attempted to demonstrate that humans are not the only ones capable of mastering grammars above FSG. These include Rey et al. (2012) for baboons, Abe and Watanabe (2011) for Bengalese finches, and Gentner et al. (2006) for starlings. See Fitch (2017) and ten Cate (2017) for critiques. There are other works that have tested whether nonhuman animals are capable of learning non-adjacent dependencies, including Newport and Aslin (2004), Newport et al. (2004), Malassis et al. (2018), and Versace et al. (2019). As Wilson et al. [(2020): 853] conclude, “these studies demonstrate that at least some nonhuman animals appear to be sensitive to these types of nonadjacent dependencies, but also point to potential cross-species differences, including between humans and nonhuman animals, in how they might be learned.”

^2^See Rendall (2021) for critique of recent work on primate semantics and syntax.

^3^Progovac (2015) proposed what she calls a “two-slot mold.” This is similar to our dual-compartment frame, although her proposal is based on speculation about earlier forms of human language.

^4^Most works aiming at eliciting an FSG grammar from nonhuman primates resort to pattern discrimination rather than on the combinatorial complexity of vocal production. There is no known case of vocal production that combines more than two calls, as observed in Miyagawa and Clarke (2019). This binarity recalls the dichotomy between vocal production/vocal perception in nonhuman primates: while the former is highly restricted, the latter is argued to be more sophisticated (Seyfarth and Cheney, 1986, 2003; Fitch and Zuberbühler, 2013). At this point, however, there is no reason to associate a full-fledged FSG to tamarins even in their pattern-discrimination perception.

^5^Kaan and Swaab (2002) and Matchin and Hickok (2016) argue that syntactic operations do not just recruit a specific frontal region such as the Broca's.

^6^In Zaccarella and Friederici (2015a,b,c), they assign to the adjacent anterior insula the role of processing two-word sequences, a brain region that is linked to the frontal operculum [(Friederici, 2017): 40–42].

^7^Other studies implicate the left anterior temporal lobe in human language combinatorial/hierarchical operations without mention of Broca's area and the frontal operculum (Bemis and Pylkkänen, 2011; Brennan and Pylkkänen, 2017).
